# Defining the Clinical, Molecular and Ultrastructural Characteristics in Occipital Horn Syndrome: Two New Cases and Review of the Literature

**DOI:** 10.3390/genes10070528

**Published:** 2019-07-12

**Authors:** Aude Beyens, Kyaran Van Meensel, Lore Pottie, Riet De Rycke, Michiel De Bruyne, Femke Baeke, Piet Hoebeke, Frank Plasschaert, Bart Loeys, Sofie De Schepper, Sofie Symoens, Bert Callewaert

**Affiliations:** 1 Center for Medical Genetics Ghent, Ghent University Hospital, 9000 Ghent, Belgium; 2 Department of Dermatology, Ghent University Hospital, 9000 Ghent, Belgium; 3 Department for Biomedical Molecular Biology, Ghent University, 9000 Ghent, Belgium; 4 VIB Center for Inflammation Research, 9000 Ghent, Belgium; 5 Ghent University Expertise Centre for Transmission Electron Microscopy and VIB BioImaging Core, 9000 Ghent, Belgium; 6 Department of Urology, Ghent University Hospital, 9000 Ghent, Belgium; 7 Department of Orthopedic Surgery, Ghent University Hospital, 9000 Ghent, Belgium; 8 Center for Medical Genetics, University of Antwerp/Antwerp University Hospital, Antwerp, Belgium

**Keywords:** occipital horn syndrome, Ehlers–Danlos syndrome type IX, Menkes syndrome, ATP7A, review, copper transport, elastic fiber, collagen

## Abstract

Occipital horn syndrome (OHS) is a rare connective tissue disorder caused by pathogenic variants in ATP7A, encoding a copper transporter. The main clinical features, including cutis laxa, bony exostoses, and bladder diverticula are attributed to a decreased activity of lysyl oxidase (LOX), a cupro-enzyme involved in collagen crosslinking. The absence of large case series and natural history studies precludes efficient diagnosis and management of OHS patients. This study describes the clinical and molecular characteristics of two new patients and 32 patients previously reported in the literature. We report on the need for long-term specialized care and follow-up, in which MR angiography, echocardiography and spirometry should be incorporated into standard follow-up guidelines for OHS patients, next to neurodevelopmental, orthopedic and urological follow-up. Furthermore, we report on ultrastructural abnormalities including increased collagen diameter, mild elastic fiber abnormalities and multiple autophagolysosomes reflecting the role of lysyl oxidase and defective ATP7A trafficking as pathomechanisms of OHS.

## 1. Introduction

Occipital horn syndrome (OHS, OMIM#304150), previously known as Ehlers–Danlos syndrome type IX or X-linked cutis laxa, is a rare disorder characterized by prominent connective tissue abnormalities including cutis laxa, hernias, joint laxity and bladder diverticula, and pathognomonic exostoses with “occipital horns” or downward pointing exostoses situated in the tendinous insertions of the sternocleidomastoid and trapezius muscles. In addition, patients may show mild to moderate intellectual disability [1,2,3,4,5,6]. Being allelic with Menkes disease (MD, OMIM#30011), OHS is considered the milder end of the phenotypic spectrum due to pathogenic variants in *ATP7A* [4] that encodes a copper transporter. Several clinical features in both disorders are related to the malfunctioning of cupro-enzymes, including lysyl oxidase, dopamine ß-hydroxylase, tyrosinase and cytochrome C oxidase [7,8,9,10].

ATP7A mediates (1) copper transport from the gastrointestinal tract into the bloodstream, (2) intracellular delivery of copper to cupro-enzymes in the secretory pathway and (3) efflux of excess copper from the cell [11,12]. Intracellular ATP7A trafficking is essential for copper homeostasis. Under basal low copper conditions, ATP7A is found in the trans-Golgi network, where it is essential for cupro-enzyme biogenesis. At higher copper concentrations, it reversibly localizes to the plasma membrane and post-Golgi compartments, where it is responsible for the extrusion of excess copper from the cell [13,14,15]. The *ATP7A* gene maps to Xq13.3 and spans a genomic region of ~140 kb. Its predominant transcription product contains 23 exons [16,17,18,19,20]. ATP7A belongs to the large P-type ATPase family, which are ATP-driven membrane pumps essential in the maintenance of electrochemical gradients (such as Na^+^/K^+^-, H^+^/K^+^- and Ca^2+^-pumps), as well as lipid and cationic homeostasis [21], including ATP7A and ATP7B, both important for copper homeostasis [13,22]. P-type ATPases share a structural core containing a transmembrane (TM) domain responsible for transport and an N-, P- and A- soluble catalytic domain, respectively involved in nucleotide binding, phosphorylation and dephosphorylation [23]. The transmembrane domain of ATP7A contains eight transmembrane helices (TM1–TM8). Six amino terminal domains contain metal-binding CXXC motifs (MBD1–MBD6) [24,25].

MD is mostly caused by truncating variants, including nonsense variants, frameshift variants and splice-site variants that lead to out of frame transcripts and large deletions, which result in very low to absent ATP7A levels [12,26]. On the contrary, the milder symptoms in OHS are the consequence of pathogenic variants in *ATP7A*, mostly “leaky” splice-site variants resulting in exon-skipping, which permit the production of a small amount of normal ATP7A mRNA [6,13,27,28,29,30]. This low amount of normal mRNA can result in residual protein activity and allow effective cation transfer to some copper-dependent enzymes. ATP7A levels as low as 2–5% have been shown to be sufficient to result in the milder OHS phenotype [31]. Connective tissue abnormalities in OHS have been attributed to a decreased activity of lysyl oxidase (LOX), a cupro-enzyme that normally deaminates lysine and hydrolysine residues in the first step of collagen crosslinking [7,32]. Patients may also encounter mild neurological signs and dysautonomia due to partial defects in dopamine-ß-hydroxylase enzyme activity, that converts dopamine to norepinephrine, a crucial neurotransmitter in norepinephrinergic neurons. As such, patients with OHS typically have low to normal levels of serum copper and ceruloplasmin together with abnormal plasma and cerebrospinal fluid (CSF) catecholamine levels [3,33,34].

Due to the rarity of OHS and the limited number of cases scattered in literature, the natural history and clinical phenotype remain incompletely studied. This study reports the clinical, molecular and ultrastructural data for two new cases of OHS. In addition, we performed a clinical review of 32 previously described patients [6,7,11,27,28,29,30,31,35,36,37,38,39,40,41,42,43,44,45,46,47,48,49,50,51].

## 2. Materials and Methods

### 2.1. Patients

**Figure 1:**
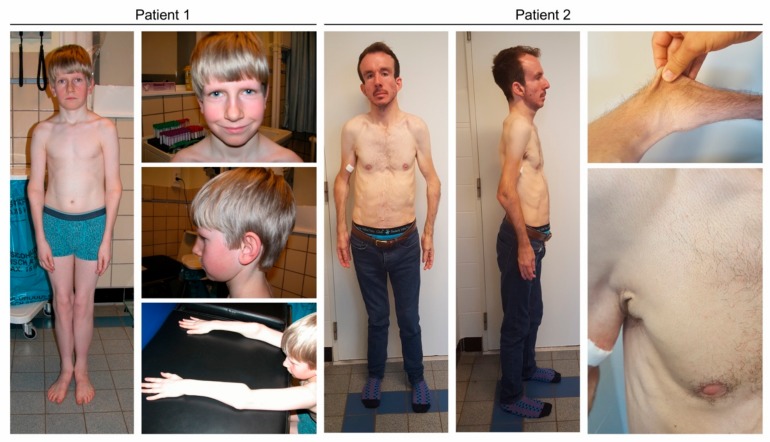
Clinical characteristics in subjects F1:II-1 and F2:II-1. Subject F1:II-1 displays mild facial dysmorphism with a flat face, deep-set eyes, a long and narrow nose, large ears and mild webbing of the neck. Craniofacial features in patient F2:II-1 are more pronounced and include dolichocephaly, a high forehead, biparietal narrowing, downslanting eyes, a convex nasal ridge, midfacial hypoplasia, micrognathia and low-set ears. No hair abnormalities were observed. Both have lax and hyperextensible skin, marked joint hypermobility and deformities of the proximal radius.

Informed consent was obtained from subject 1 and his parents, and from subject 2. Additional consent was given to publish the clinical pictures in Figure 1. Clinical information was obtained by evaluating both patients at our center with the use of a detailed clinical checklist. We performed a literature review of 32 OHS patients with confirmed *ATP7A* pathogenic variants in the Pubmed and Embase databases, only including studies with ample clinical data. Selection of the reports was undertaken independently by two different authors (A.B and K.V.M). Clinical data from the literature were evaluated using the same methodology (Supplementary Table S1). This study was conducted in accordance with the Declaration of Helsinki and approved by the Ghent University Hospital ethics committee (registration number B670201319316).

#### 2.1.1. Clinical Reports

##### Case 1

Subject 1 (F1:II-1) was born at term after an uncomplicated pregnancy. At birth, his weight was 2400 g, length 45.5 cm and head circumference 32.4 cm. Developmental milestones were achieved normally. At three years of age, he presented with micturition problems evolving to urinary retention, which led to the discovery of giant bladder diverticula. Over subsequent years, several diverticulectomies were performed, followed by continent diversion and intermittent catheterization.

Clinical examination at 11 years of age showed mild facial dysmorphism (Figure 1) with a flat face, deep-set eyes, a long narrow nose, large ears and mild webbing of the neck. His weight was 24.9 kg (P1, −2.37 SD), height 139.2 cm (P26, −0.649 SD) and head circumference 56.2 cm (P98, +2.1 SD). Skeletal abnormalities included joint hyperlaxity of the wrists, metacarpophalangeal joints and fingers, a flat back, marked bony thickening of the proximal radius and tibia, genua valga and pedes plani. There was skin webbing between the second and third toe. His skin was soft and mildly hyperextensible with fine wrinkles over the abdomen and dorsum of hands and feet. He had no hair abnormalities. Neurological examination was normal. He had recurrent inguinal hernias. He reported bruising easily, but no impaired wound healing. He suffered from chronic joint pain. The patient attended normal education.

**Figure 2:**
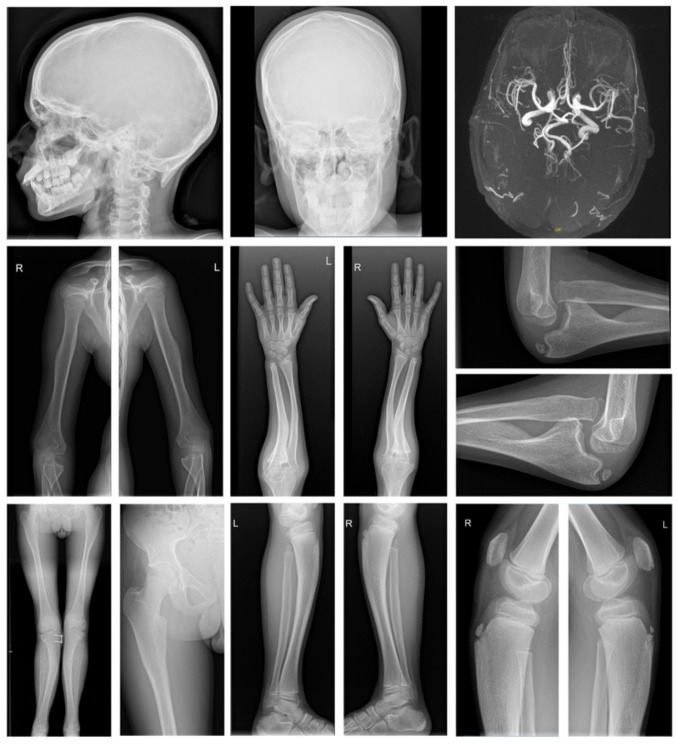
Total skeletal radiography and MRI/MRA studies in subject F1:II-1 showing severe skeletal dysplasia with occipital horns, undertubulation of bony structures and broadening of the ventral end of the first left rib, both claviculae and scapular neck, bowing of the long bones with mid-diaphyseal broadening, bilateral luxation of the radial heads, bilateral overgrowth of the ulnar coronoid processes, prominent trochanter minor of both femurs, short fibula, bilateral coxa valga with rounded iliac wings and broad metatarsals. Brain MRI and angiography showed tortuous intracranial arteries and normal brain structure.

Additional examinations included a total skeletal radiography and MRI/MRA (Figure 2). X-rays show severe skeletal dysplasia, occipital horns, undertubulation of bony structures and broadening of the ventral end of the first left rib, the distal end of both claviculae and the scapular neck. The long bones showed bowing with mid-diaphyseal broadening. There was bilateral luxation of the radial heads, bilateral overgrowth of the ulnar coronoid processes and prominent trochanter minor of both femurs. We observed a short fibula and bilateral coxa valga with rounded iliac wings. The metatarsals were broad. The vertebrae showed platyspondyly. Brain MRI and angiography showed tortuous intracranial arteries. Echocardiography, lung radiography and spirometry were normal. Serum copper and ceruloplasmin levels were within normal ranges (89.6μg/dL (70–140) and 0.287 g/L (0.2–0.6), respectively).

##### Case 2

Subject 2 (F2:II-1) was evaluated at the age of 30. He was born at term after an uncomplicated pregnancy. He developed an inguinal hernia at six weeks of age. Since then, he had multiple inguinal and femoral hernias requiring repeated mesh repairs. Starting from two years of age, he had severe bladder problems, including recurrent urinary tract infections and urinary retention, due to multiple bladder diverticula. After more than 20 surgical bladder repairs, he started self-catheterization at 8 years of age. Aged 20 years, he underwent left nephrectomy for a non-functioning kidney. He reported a mild motor delay and walked at two years of age. Joint hypermobility resulted in repeated shoulder and elbow subluxations. He was of normal intelligence and pursued higher education. He suffered from migraine, worsening musculoskeletal pain, fatigue, and depression for which he was treated with gabapentin and amitriptyline. He had a history of mitral valve prolapse, orthostatic hypotension, palpitations and a prolonged QT interval on electrocardiogram. Finally, he had a longstanding history of upper and lower gastrointestinal symptoms including nausea, post-prandial vomiting and chronic diarrhea, possibly in the context of dysautonomia. He was diagnosed with asthma.

His height was 169 cm, his weight was 50.6 kg and his head circumference was 58.5 cm. Craniofacial features consisted of dolichocephaly, a high forehead, biparietal narrowing, downslanting eyes, a convex nasal ridge, midfacial hypoplasia, micrognathia and low-set ears. He had lax and hyperextensible skin (Figure 1). He had no hair abnormalities. Skeletal abnormalities included occipital horns, a loss of sagittal spine curvature, scoliosis, pectus excavatum, and restricted elbow extension. He had bowing of the ulna and radius, bilateral exostoses of the radii and tibiae, bilateral rounding of the iliac wings, cranial displacement of the right humeral head and genua vara. Neurological examination and brain MRI were normal. Serum copper and ceruloplasmin levels were 8.3 μmol/L (11–20) and 0.13 g/L (0.20–0.50), respectively.

### 2.2. Molecular Analysis

For individual 1, genomic DNA was extracted from blood leukocytes using standard procedures. Array comparative genomic hybridization (180 k arrayCGH, Agilent Technologies, Santa Clara, CA, USA) showed a 300 kb deletion of chromosome band 1q31.1 (1q31.1.1q31.1(188407205-188707983)x1)(hg19/GRCh37), which does not contain any known genes. *FBLN5* (OMIM#219100, GenBank RefSeq accession number NM_006329.3), *LTBP4* (OMIM#613177, NM_003573.2) and *ATP7A* (OMIM#304150, NM_000052.7) gene sequencing was performed without the identification of a pathogenic mutation. Total RNA was extracted from cultured fibroblasts using the RNeasy Kit (Qiagen, Hilden, Germany) and cDNA was synthesized with the iScript cDNA Synthesis Kit (Bio-Rad Laboratories, Hercules, CA, USA). *ATP7A* cDNA analysis was performed followed by confirmation with long-range RT-PCR and next-generation sequencing (Miseq, Illumina, San Diego, CA, USA). For individual 2, array comparative genome hybridization was normal after which *ATP7A* gene sequencing (GenBank RefSeq accession number NM_000052.7) was performed.

### 2.3. Transmission Electron Microscopy (TEM)

For transmission electron microscopy, 3 mm skin fragments from both individuals and an age- and sex-matched control were immersed in a fixative solution of 2.5% glutaraldehyde and 4% formaldehyde in Na-Cacodylate buffer 0.1 M, placed in a vacuum oven for 30 min and left rotating for 3 hours at room temperature. This solution was later replaced with fresh fixative and samples were left rotating over night at 4 °C. After washing, samples were post-fixed in 1% OsO_4_ with K_3_Fe(CN)6 in 0.1 M NaCacodylate buffer, pH 7.2. After washing in double-distilled H_2_O, samples were subsequently dehydrated through a graded ethanol series, including bulk staining with 2% uranyl acetate at the 50% ethanol step, followed by embedding in Spurr’s resin. To select the area of interest on the block and in order to have an overview of the phenotype, semi-thin sections were first cut at 0.5 µm and stained with toluidine blue. Ultrathin sections of a gold interference color were cut using an ultramicrotome (Leica EM UC6, Wetzlar, Germany), followed by post-staining in a Leica EM AC20 for 40 min in uranyl acetate at 20 °C and for 10 min in lead stain at 20 °C. Sections were collected on formvar-coated copper slot grids. Grids were viewed with a JEM 1400plus transmission electron microscope (JEOL, Tokyo, Japan) operating at 80 kV.

### 2.4. Collagen Biochemical Analysis

Fibroblast cultures were started from skin biopsies from individuals 1 and 2, and a matched control. Biochemical (pro)collagen sodium dodecyl sulfate polyacrylamide gel electrophoresis (SDS-PAGE analysis) was performed on the medium and cellular fractions of the cultured skin fibroblasts. When confluent, cells were labeled with 14C-proline [52]. After SDS-PAGE separation, further processing of the gels was performed as described previously by our group [53].

## 3. Results

### 3.1. Clinical Characteristics

We describe the clinical characteristics of two new individuals with OHS and 32 patients described in the literature (Table 1, Supplementary Table S1). All patients were male, except for patient F17:II-2. The mean reported age of patients was 19.6 years (mean 17, range 1–50). Disease-related mortality was observed in 5 out of 34 patients. The mean age at death was 21.2 years and causes included respiratory failure (post-surgery apnea), gastric perforation and diffuse intravascular coagulation due to a large cephalhematoma at birth.

Initial presentations leading to the diagnosis are presented in Table 2. One-third of patients presented with neurological features including hypotonia, developmental delay, and seizures. Nine individuals presented with connective tissue problems such as cephalhematoma and inguinal hernia. Other presenting symptoms included bladder problems such as bladder diverticula, urinary tract infections and skeletal problems.

Patients displayed variable craniofacial features, including a long face (6/13, 46%), large ears (5/13, 38%), sagging cheeks (5/11, 45%) and hair abnormalities (14/19, 74%). Trichoscopy confirmed pili torti in 7 out of 10 examined individuals. Connective tissue abnormalities were reported in all subjects. Cutis laxa was present in the majority of patients (25/26, 94%), as well as inguinal hernias (15/25, 63%). An umbilical hernia and hiatal hernia were observed in only one patient each (F25:II-1 and F15:II-1, respectively). Wound healing was reported to be normal after trauma or surgery. Occipital horns were reported in nearly all cases (25/26, 96%). Other recurrent skeletal manifestations included hammer-shaped claviculae (9/19, 47%), scoliosis (8/21, 38%), pectus deformities (13/23, 57%), radial/tibial exostoses (6/16, 38%), coxa valga (6/16, 38%) and genua valga (6/17, 35%). Dislocations, mainly of the radial heads and joint hypermobility were present in about two-thirds of individuals, 12/20 (60%) and 16/26 (62%), respectively. Less frequently reported skeletal manifestations include bowing of the long bones, mid-diaphyseal broadening, metaphyseal spurring and rounding of the iliac wings. Osteopenia or osteoporosis was reported in four patients and fractures were observed in two subjects.

Mild, mainly motor, developmental delay, was observed in 17 subjects (17/30, 57%). Intellectual disability was present in an equal number of patients (17/33, 52%) of whom seven had mild, four had moderate and three had severe intellectual disability. In three patients, the severity of intellectual disability was not specified. Several subjects displayed muscle hypotonia, especially at a young age (before the age of two). Seizures were documented in five patients (5/25, 20%). One individual was diagnosed with a spontaneous intracerebral hemorrhage at the age of 15, resulting in aphasia and spastic quadriparesis. MRI brain studies were performed in 11 patients and documented to be abnormal in 7 subjects, showing mild atrophy and/or delayed myelination. Intracranial tortuosity was present in two-thirds of patients (7/11, 64%). Tortuosity of extracranial arteries was found in three cases and was located in the cervical, splenic and hepatic arteries. Aneurysm formation was found in five patients, mainly affecting the same arteries. One individual was diagnosed with a type A aortic dissection at the age of 39, which was successfully surgically manage. Two patients showed dilatation of the large veins. Orthostatic hypotension, temperature instability, chronic diarrhea, and other symptoms of dysautonomia were seen in a high number of patients (13/15, 87%). Severe urological complications are an important concern in OHS, with bladder diverticula in 83% (25/30) of reported patients, of which three were eventually diagnosed with a bladder rupture. Recurrent urinary tract infections based on incomplete emptying of the bladder were seen in 74% (17/23) of patients. One-third of patients had vesicourethral reflux (VUR), sometimes complicated by secondary renal problems. Asthma was reported in four cases. Low levels of serum copper and ceruloplasmin were found in two-thirds of cases, 20/28 (71%) and 20/27 (74%), respectively.

### 3.2. Molecular Characteristics

After normal *ATP7A* gene sequencing, cDNA analysis showed a deletion of exon 10 in subject F1:II-1. Subsequent confirmation with long-range RT-PCR revealed a deep intronic c.2407-516G>T variant, underlying the exon 10 skipping, likely due to the activation of a cryptic splice acceptor. In patient F2:II-1, *ATP7A* gene sequencing identified a hemizygous c.2917-4A>G splice-site variant in *ATP7A*.

**Figure 3:**
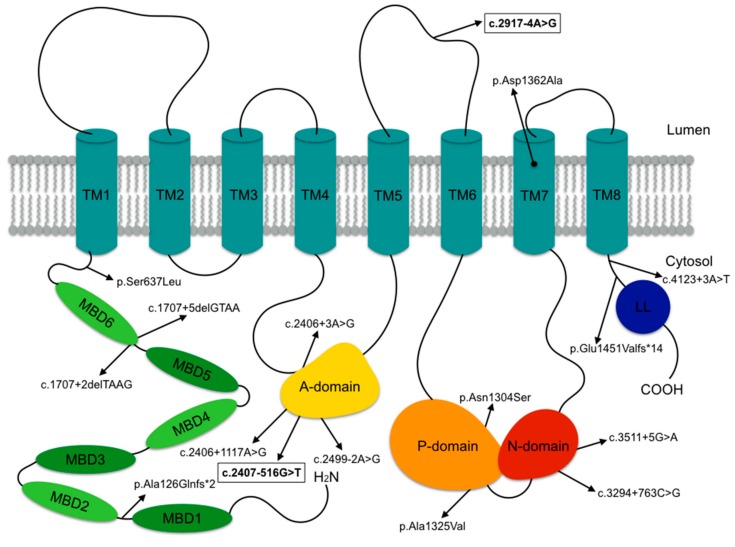
ATP7A pathogenic variants reported in this study. ATP7A consists of six amino terminal metal binding domains (MBD1-MBD6), a transmembrane domain containing eight transmembrane helices (TM1-TM8) and an N-, P- and A- soluble catalytic domain. The pathogenic variants identified in subjects F1:II-1 and F2:II-1 are indicated in bold and outlined.

A total of 16 different *ATP7A* pathogenic variants were reported (Figure 3); these included seven splice-site, four missense and two frameshift variants, two deep intronic variants and one deletion. In eight patients, the specific variant was not reported, including a 98-bp deletion in the regulatory region (F9:II-1), a deep intronic intron six splice donor site variant (F12:II-1) and a duplication in exon 11 and 12 (F23:II-1). Nonsense ATP7A variants were not observed. Pathogenicity information and CADD scores can be found in Supplementary Table S2. We did not observe any clear genotype-phenotype correlation and there was no correlation with serum copper and ceruloplasmin levels (Supplementary Table S3).

### 3.3. Transmission Electron Microscopy

**Figure 4:**
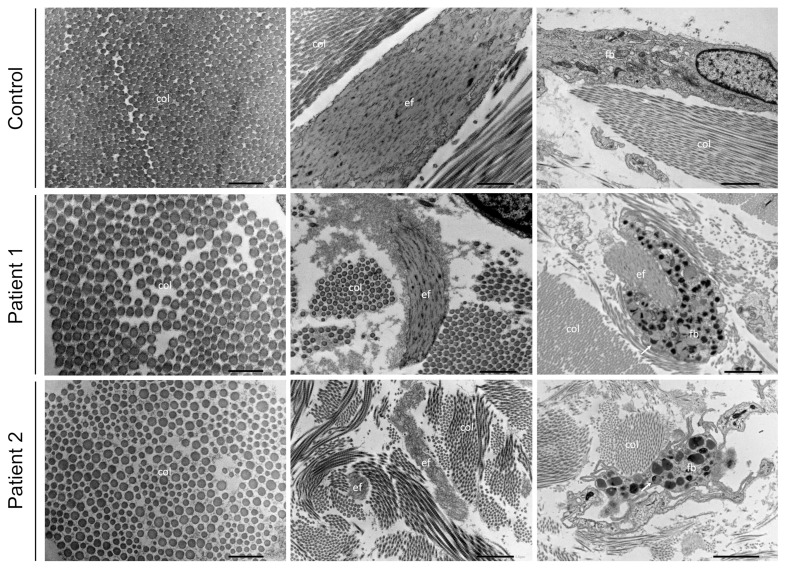
Transmission electron microscopy findings in occipital horn syndrome. Transmission electron microscopy (TEM) findings in skin samples of patients F1:II-1 and F2:II-1 and a matched control showing collagen (column 1), elastic fibers (column 2) and a fibroblast (column 3). col = collagen; ef = elastic fiber; fb = fibroblast. Magnification for column 1: ×15,000, column 2: ×8000 and column 3: ×4000.

Transmission electron microscopy (TEM) findings in skin samples of subjects F1:II-1, F2:II-1 and a matched control are shown in Figure 4. Collagen fibrils in the dermis of the control sample are regularly organized in curvilinear bundles with uniform diameters. In the dermis of both OHS patients, the collagen fiber diameter appears to be larger and more variable. The fibrils are more loosely packed (especially in F2:II-1) but retain a normal shape. TEM of a control elastic fiber demonstrates a solid and dense core of elastin surrounded by a sparse mantle of microfibrils. In both individuals, the elastin core is surrounded by a debris extending outside the elastic fiber with loss of the microfibrillar organization. In addition, the elastin core in subject F2:II-1 is almost completely disrupted and difficult to discriminate. Fibroblast abnormalities are observed in both patients, with multiple autophagolysomes containing sickle-shaped electron dense deposits (Supplementary Figure S1). The mitochondria, Golgi complex, and endoplasmatic reticulum appear normal.

### 3.4. Collagen Biochemistry

Considering the collagen abnormalities observed in both individuals with transmission electron microscopy, SDS-PAGE analysis of the intracellular and secreted (pro)collagen proteins produced by skin fibroblasts was performed for subjects 1 and 2 and a matched control. In both cases, we observed normal SDS-PAGE migration patterns (Supplementary Figure S2).

## 4. Discussion and Conclusions

The absence of large case series and natural history studies precludes efficient diagnosis and management of OHS patients. This study describes the clinical and molecular characteristics of two new patients and 32 patients previously reported in the literature. OHS is considered a mild but rare allelic variant of Menkes disease. OHS patients show prominent connective tissue abnormalities and have a better prognosis and survival rate than patients with MD [3], with low childhood mortality (1/34) in OHS. However, there is still significant mortality in (young) adults, often related to gastrointestinal, respiratory, or bleeding complications, such as an intestinal perforation due to a gastric ulcer and (post-surgery) apnea.

The clinical characteristics of OHS are listed in Table 2. Craniofacial features include mild facial dysmorphism with a long face, large ears and sagging cheeks in about half of reported patients. Hair abnormalities, including coarse hair and pili torti, are frequently present. Ubiquitous connective tissue manifestations in OHS include increased skin laxity and inguinal hernia.

Concerning the skeletal features, the pathognomonic occipital horns are present in all patients but one, which is probably an age-dependent discrepancy [41,54,55]. Other less specific skeletal abnormalities include hammer-shaped claviculae and radial/tibial exostoses. Scoliosis, pectus deformities, coxa and genua valga, joint luxations, mainly of the radial heads and general joint hypermobility are frequent but non-specific. These skeletal problems may limit daily activities and often require multiple surgical interventions.

Urological complications, mostly giant bladder diverticula and vesicourethal reflux, are found in over 80% of subjects. Secondary problems such urinary tract infections, urinary retention, bladder rupture, or VUR-mediated renal failure, which are all related to the bladder diverticula, are of great concern. Surgical interventions removing the diverticula often fail, with frequent relapse of the diverticula. Self-catheterization, vesicostomy, or continent diversion are often required for proper emptying of the bladder.

Previous reports describe a mild neurological phenotype limited to slight generalized muscle weakness and symptoms of dysautonomia [3,27], however, more detailed case studies reveal some conspicuous observations including developmental delay and intellectual disability in two-thirds of subjects and documented seizures in five individuals. Nevertheless, neurological affliction remains rather mild and normal intelligence is possible. Symptoms of dysautonomia, including chronic diarrhea, temperature instability, and orthostatic hypotension are present in almost 90% of patients and can be disabling.

Brain imaging studies are rarely performed in OHS patients and are often reported as normal. However, intracranial arterial tortuosity are noted in approximately two thirds of patients. Extracranial arterial tortuosity is less frequently observed, but has been reported in the cervical, splenic and hepatic vasculature. Aneurysm formation may complicate pre-existing tortuosity and may affect the arterial and venous circulation.

OHS is often diagnosed upon the identification of connective tissue anomalies, including cutis laxa, joint hypermobility with hypotonia, mild intellectual impairment and bladder diverticula. Later on, the pathognomonic occipital horns become more evident. The initial differential diagnosis includes other forms of cutis laxa, including *FBLN5*- and *LTBP4*-related cutis laxa (OMIM#219100 and #613177) [56] and the dermatosparaxis type of Ehlers–Danlos syndrome (EDS type VIIC, OMIM#225410) [57], which may present with cutis laxa, hypotonia and bladder diverticula, but with normal mental development. However, in OHS there is no early onset emphysema as in *FBLN5*- and *LTBP4*- related cutis laxa, nor easy bruising or severe skin fragility as in the dermatosparaxis type of EDS. *ATP6V0A2*-related cutis laxa (OMIM#219200) may also present with mild intellectual impairment but does not commonly show bladder diverticula or exostoses. Later on the exostoses can be mistaken for hereditary multiple exostoses due to defects in *EXT1* or *EXT2* (OMIM#133700 and #133701) [58].

Concerning follow-up, routine evaluation of neurodevelopment and early intervention with physiotherapy is recommended, as well as regular urological evaluation, including video urodynamic studies of bladder function. Any pain, or vascular or neurological symptoms that may relate to exostoses should be promptly investigated. To date, there are no data on a possible risk for chondrosarcomas in exostoses although these have been described in hereditary multiple exostoses [59]. It could be recommended to perform MR angiography and echocardiography from puberty onwards. Indeed, aortic root dilatation and dissection, and an increased risk for intracranial bleeds and ischemic complications have been described in other related connective tissue disorders such as Ehlers–Danlos, arterial tortuosity, Marfan and some cutis laxa syndromes. Furthermore, spirometry might detect asthma at an early stage as it was reported in four individuals. Prolonged vigilance for apnea is necessary following surgery.

The molecular data confirmed that pathogenic variants in *ATP7A* causing OHS mainly included splice-site variants, while missense and frameshift variants were less frequently observed. This observation may fit the hypothesis that a low amount of normal *ATP7A* mRNA and residual ATP7A functioning is sufficient to prevent the more severe MD [13,27,29,31,60]. No mutational hot spots were observed (Figure 3). Importantly, nearly all pathogenic variants are located in the endofacial domains and only two affect a transmembrane domain or cytosolic loop, respectively. This highlights the critical role of the transmembrane domains forming the channel for copper translocation [13]. Variants in OHS mainly affect residues implicated in ATPase-coupled copper transport. Some of these variants are predicted to inhibit the catalytic dephosphorylation leading to permanent distribution of the protein to the plasma membrane and cytosolic vesicles [14,15,21,61,62].

Our transmission electron microscopy data in skin samples of OHS individuals show larger diameters of collagen fibrils that were more loosely packed compared to those of the control sample. Elastic fibers were affected to a lesser extent but are fragmented and/or surrounded by extracellular matrix debris. OHS is associated with decreased activity of the copper dependent lysyl oxidase (LOX), that oxidates lysyl and hydroxylysyl residues in collagen and lysyl residues in elastin to aldehydes [7]. These aldehyde residues are than condensed to form cross-links, essential for collagen fibril stabilization and the integrity and elasticity of mature elastin [32,63]. Overall, this would not affect the amount or migration of soluble collagens from the medium or cellular fraction upon electrophoresis but will affect the assembly in the extracellular matrix. Also, lysyl oxidase may form cross-links with the N-propeptide domain of collagen type V, essential in collagen diameter regulation [64,65]. Fibroblasts synthesize only low amounts of type V collagen which is difficult to evaluate by electrophoresis [66,67]. Indeed, irregular fibril diameters are reminiscent of classic Ehlers–Danlos syndrome, despite the absence of cauliflower fibrils [68].

The observation of major fibroblast abnormalities, with multiple autophagolysosomes containing sickle-shaped electron dense deposits, is novel and might point to defective trafficking and trapping of proteins needing cuproenzyme-dependent processing in the post-Golgi network [14,15,21].

In conclusion, this study sheds light on the natural history of OHS by describing two new patients and 32 patients reported in the literature. Mortality in adulthood warrants long-term specialized care and follow-up, incorporating MR angiography, echocardiograpy and spirometry into standard follow-up guidelines for OHS patients, next to orthopedic and urological follow-up. Ultrastructural abnormalities including increased collagen diameter, mild elastic fiber abnormalities, and multiple autophagolysosomes reflect the role of LOX and defective ATP7A trafficking in the pathomechanism of OHS.

## Figures and Tables

**Table 1:** Overview of the phenotypic presentation in occipital horn syndrome.

	Total (Percentage)
**Craniofacial**	
Long face	6/13 (46%)
Large ears	5/13 (38%)
Sagging/ full cheeks	5/11 (45%)
Hair abnormalities	14/19 (74%)
Pili torti on trichoscopy	7/10 (70%)
**Connective tissue**	
Cutis laxa	25/27 (93%)
Inguinal hernia	15/24 (63%)
Umbilical hernia	1/22 (5%)
**Osteoarticular**	
Occipital horns	25/26 (96%)
Radial/tibial exostoses	6/16 (38%)
Hammer-shaped clavicula	9/19 (47%)
Bowing of long bones	4/17 (23%)
Mid-diaphyseal broadening	2/17 (11%)
Rounding of the iliac wings	3/17 (18%)
Coxa valga	6/16 (38%)
Genu valgum	6/17 (35%)
Metaphyseal spurring	2/16 (13%)
Scoliosis	8/21 (38%)
Pectus deformity	13/23 (57%)
Dislocations	12/20 (60%)
Contractures of large joints	3/19 (16%)
Joint hyperlaxity	16/26 (62%)
Fractures	2/21 (10%)
**Neurological**	
Intellectual disability	17/33 (52%)
Seizures	5/25 (20%)
Muscle hypotonia	10/25 (40%)
Stroke	1/23 (4%)
**Cardiovascular**	
Aneurysm formation	5/7 (71%)
Dilatation of the large veins	2/6 (33%)
Intracranial tortuosity	7/11 (64%)
Extracranial tortuosity	3/4 (75%)
Dysautonomia	13/15 (87%)
**Urogenital**	
Bladder diverticula	25/30 (83%)
Renal abnormalities	6/20 (30%)
Urinary tract infections	17/23 (74%)
Vesicourethral reflux	7/23 (30%)
**Laboratory findings**	
Serum copper	20/28 (71%)
Serum ceruloplasmin	20/27 (74%)

**Table 2:** Initial presentations leading to the diagnosis in occipital horn syndrome.

Initial Presentation	Number of Patients
**Neurological**	**11**
Seizures	1
Developmental delay	4
Hypotonia	6
**Connective tissue**	**9**
Cephalhematoma	4
Generalized CTD	3
Inguinal hernia	2
**Urogenital**	**5**
Bladder diverticula	3
Urinary infections	2
**Skeletal**	**3**
Pectus deformity	1
Skeletal dysplasia	1
Joint pain	1
**Other**	**3**
Vomiting and diarrhea	1
Dysautonomia	1
Apnea	1
**Segregation analysis**	**1**
**Unknown**	**2**

“CTD”: connective tissue disease.

## Supplementary Materials

The following are available online at https://www.mdpi.com/2073-4425/10/7/528/s1; Supplemental Figure S1: Fibroblast abnormalities in OHS. Magnification: x4000, x8000 and x15000; Supplemental Figure S2: SDS-PAGE collagen analysis in patients F1:II-1 and F2:II-1 and a matched control. Panel A: medium procollagen fraction on fibroblasts in culture for three days. Panel B: cellular collagen fraction; Supplemental Table S1. Clinical characteristics of all patients. ‘”+”: present, “-“: absent, “M”: male, “F”: female, “y”: year(s), “mo”: month(s), “d”:day(s), “CVA”: cerebrovascular accident, “DIC”: diffuse intravascular coagulation, “FTT”: failure to thrive, “GER”: gastro-esophageal reflux, “SC”: subcutaneous, “UTI”: urinary tract infection; Supplemental Table S2. Pathogenicity information for the ATP7A variants in this study. The pathogenicity classification used in this table is based on the Alamut® mutation analysis software, which uses the following prediction tools: Align GVGD (Huntsman Cancer Institute, University of Utah) combines the biophysical characteristics of amino acids and protein multiple sequence alignments to where missense substitutions in genes of interest fall from enriched deleterious to enriched neutral. SIFT predicts whether amino acid substitution affects protein function. The MutationTaster prediction program integrates information from different biomedical databases, evolutionary conservation, splice-site changes and loss of protein features. Protein conservation is based on MARRVEL® multiple protein alignment. The splice prediction predicts the chance of variants having an impact on alternative splicing. Gnomad allele frequencies and Clinvar data can also be found. In the last column, CADD scores are depicted (Kircher et al., 2014). “NS”: not specified, “NA”: not applicable; Supplemental Table S3. Copper and ceruloplasmin levels in all patients.
