# Supplementary material for: Defining the Clinical, Molecular and Ultrastructural Characteristics in Occipital Horn Syndrome: Two New Cases and Review of the Literature
Source: Genes (Basel). 2019 Jul 12;10(7):528. doi: 10.3390/genes10070528 (PMC6678539; doi:10.3390/genes10070528)
Supplement: Supplementary file 1 [file genes-10-00528-s001.zip › OHS supplemental figures revision/Supplemental figure 1 OHS.pdf]

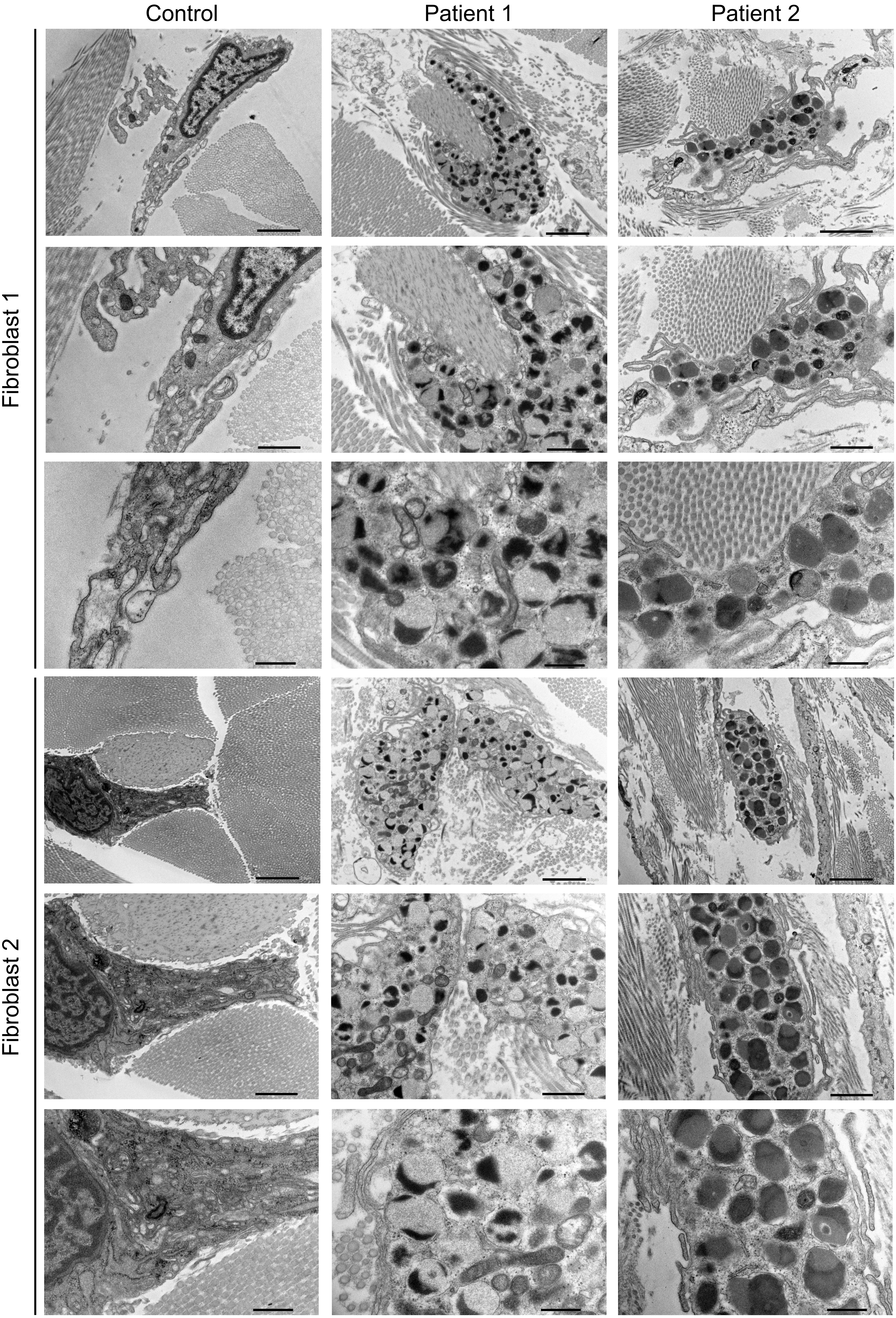

Control
Patient 1
Patient 2
Fibroblast 1
Fibroblast 2
