# Supplementary figures and images for: Defining the Clinical, Molecular and Ultrastructural Characteristics in Occipital Horn Syndrome: Two New Cases and Review of the Literature
Source: Genes (Basel). 2019 Jul 12;10(7):528. doi: 10.3390/genes10070528 (PMC6678539; doi:10.3390/genes10070528)

## A. Medium Procollagen Fraction

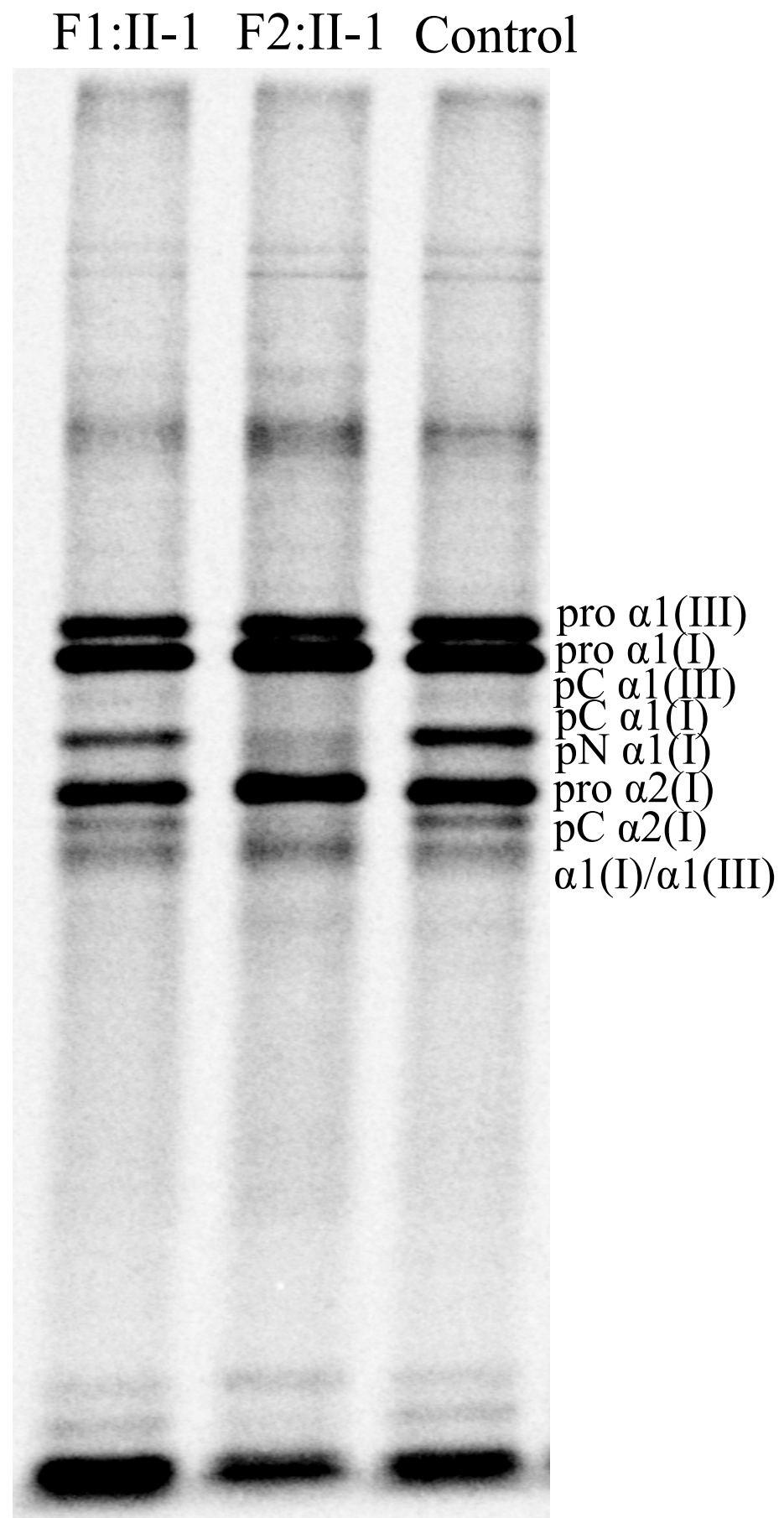

## B. Cellular Collagen Fraction

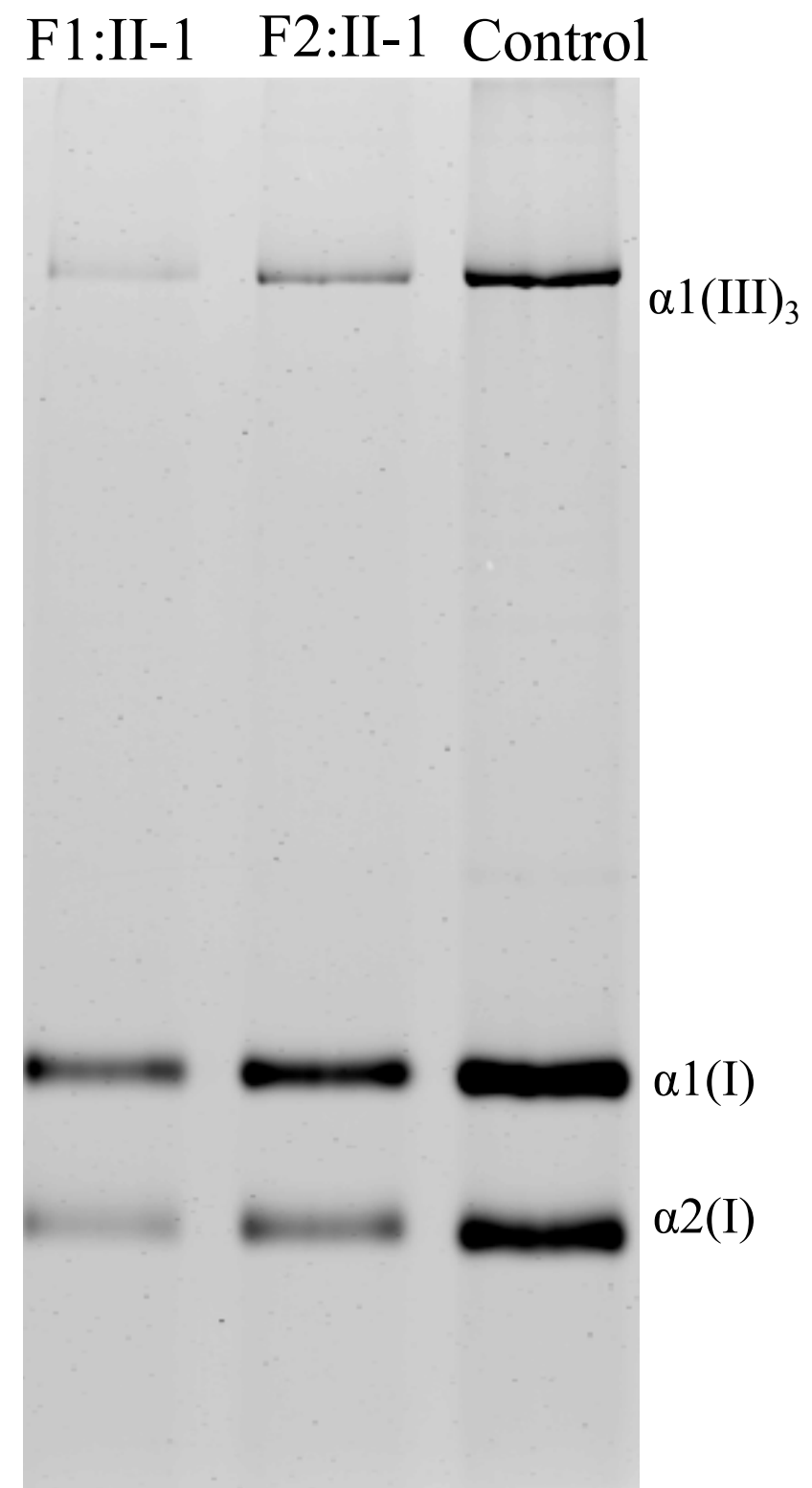

Supplement: Supplementary file 1 [file genes-10-00528-s001.zip › OHS supplemental figures revision/Supplemental figure S2 OHS.pdf]
